# Construction Notes

**Published:** 1893-04-08

**Authors:** 


### CONSTRUCTION NOTES.

It is proposed to erect a Cottage Hospital for Wrington Vale. The committee have met with a hearty response to the circulars sent out asking for donations for the same—£500 having already been received in that manner. Annual subscriptions amounting to over £47 have been promised, and £233 is in the hands of the building committee, a sum of money which was left over, and has thus accumulated from the funds of the old hospital.

At the annual meeting of the Birmingham General Hospital the committee reported that the erection of the new hospital was being pushed on as fast as possible. The site had been acquired and paid for. Operations had been somewhat delayed by the adoption of a different system of ventilation and heating from that which had been proposed by the architect. The system now to be applied was the one at work in the Victoria Hospital, Glasgow, the air being thus renewed in the wards six times an hour. The amount already promised to the building fund was £93,032, but more would be needed. In the new hospital there will be 330 beds, instead of 271 as in the old one.

The convalescent home at Costorphine of the Edinburgh Royal Infirmary, to which two wings have recently been added by the munificence of Mr. James Nasmyth, was formally opened by the Marchioness of Tweeddale on the 15th inst. Each wing contains a large day-room in the front, behind which on the male side is a smoking-room, and on the female side a private ward. At the junction of the new wings with the old buildings, new lavatories and other offices are erected. The second floor is given up to two large dormitories, containing in both cases twenty beds, each of which has twenty cubic feet space. Bath-room accommodation is afforded in connection with the new wards. A glass-roofed verandah and an entrance-porch has been added to the main block. The new wing will give accommodation for forty additional patients, the home being planned to contain 100 in all. The cost has been about £8,000, and the architects are Messrs. Kinnear and Peddie, of Edinburgh.

The committee of the building fund for the proposed new infirmary in connection with the Bridgnorth and South Shropshire Infirmary report an offer of an acre of ground near the North Gate, granted by Mr. T. Martin Southwell, as a site. The trustees of the building fund have already £303 19s. in hand, and Mr. W. O. Foster has further contributed £1,000 towards the same. It is the opinion of the committee that the cost of the building as at present projected will not fall short of, or much exceed the sum of £4,000.

Through the munificent gift of Mr. E. A. Pearn, of Tavistock, a convalescent home is to be erected at Plymouth for the benefit of the patients of the South Devon and East Cornwall Hospital in that town. Mr. Charles King, of Plymonth, has supplied the designs for the building, which will be in the Italian style of architecture, fronting due south. Two separate entrances for male and female patients are to be provided for, and a glazed verandah for sitting or walking about in. A large assembly hall will be shared by the inmates. There will be accommodation for thirteen beds in four wards, whilst there is a provision of four beds on the ground floor for such patients as are old or infirm. The building will further include all necessary offices, and rooms for the superintendent and her assistants. The establishment will be known as the Pearn Convalescent Home.

It has been decided to erect an infectious disease hospital at Hereford. The city and the sanitary surveyors received instructions to visit several institutions of a like kind, with a view to observing the size and general arrangement of such buildings. The sanitary authority having accepted a suitable plan, they have given instructions for the work to be proceeded with forthwith, at a cost, not exceeding £600, which work will be carried out by Messrs. Humphreys and Co., builders, of Knightsbridge, London. The main building will consist of two wards, with separate entrances, each ward measuring 20 feet by 30 feet, containing six beds respectively. A fair-sized convalescent ward, two bath-rooms, and a matron's-room, and the usual offices will complete the building. By means of the sliding sashes which have been introduced into the plan the main body of the building can be cut off from the wards if it is necessary to do so.

The new Isolation Hospital at Norwich was formally opened the third week of March. The site consists of four acres of land enclosed in an iron fence, and immediately adjoins the iron hospital already existing. The new building was erected from plans designed by the city architect, Mr. Winter; it consists of five blocks, all of which are constructed on one storey, with the exception of the administration block. This provides accommodation for the matron, the day nurse, the doctor, and medical officer. There are six bed-rooms for nurses and attendants, a bath-room and two lavatories. The central block provides four wards, each containing accommodation for two patients. The outer blocks contain two ward pavilions, for the accommodation of six patients, the wards being divided off by the nurses duty-room, the lobby, and bath-room. The laundry-block contains the laundry and wash-house, also a mortuary, a coach-house, and disinfecting-room. The buildings have all been arranged with the latest improvements in sanitary respects. The builders are Messrs. Wilkins, of Norwich.

The new Memorial Hospital at Mirfield is approaching its completion. This building, which is a handsome one, owes its existence to the bounty of Mr. Chas. Wheatley, of Sands House, who planned it in memory of his sister. The central front recedes somewhat, a large ward on either side projecting, thus allowing of a covered connecting veranaah. The men's ward, as also the women's, are well-lighted and ventilated rooms, 32 feet wide. Another special ward and the nurses' room are on the south side. The large operating theatre is provided with all necessary requirements and complete offices. In the grounds is a well-fitted laundry, as also a mortuary. The water supply pipes throughout the building are of the newly-patented kind, *i.e.*, iron outside and lined with uncorrodable tin. Mr. Charles Wheatley has endowed the hospital with £100 yearly. The committee, though they have power to modify and meet exceptional cases, are empowered to charge each patient the sum of 1s. per diem during their stay in the wards. The hospital was declared to be ready for opening on Easter Monday. The architect was Mr. A. A. Stott; the builders, Messrs. Fothergill and Schofield.

